# Animals in the Zika Virus Life Cycle: What to Expect from Megadiverse Latin American Countries

**DOI:** 10.1371/journal.pntd.0005073

**Published:** 2016-12-22

**Authors:** Marina Galvão Bueno, Nádia Martinez, Lívia Abdalla, Claudia Nunes Duarte dos Santos, Marcia Chame

**Affiliations:** 1 Fundação Oswaldo Cruz, Programa Institucional Biodiversidade e Saúde, Rio de Janeiro, Brazil; 2 Laboratório de Virologia Molecular, Instituto Carlos Chagas, Fundação Oswaldo Cruz, Curitiba, Paraná, Brazil; University of Queensland, AUSTRALIA

## Abstract

Zika virus (ZIKV) was first isolated in 1947 in primates in Uganda, West Africa. The virus remained confined to the equatorial regions of Africa and Asia, cycling between infecting monkeys, arboreal mosquitoes, and occasionally humans. The ZIKV Asiatic strain was probably introduced into Brazil in or around late 2013. Presently, ZIKV is in contact with the rich biodiversity in all Brazilian biomes, bordering on other Latin American countries. Infections in Brazilian primates have been reported recently, but the overall impact of this virus on wildlife in the Americas is still unknown. The current epidemic in the Americas requires knowledge on the role of mammals, especially nonhuman primates (NHPs), in ZIKV transmission to humans. The article discusses the available data on ZIKV in host animals and issues of biodiversity, rapid environmental change, and impact on human health in megadiverse Latin American countries. The authors reviewed scientific articles and recent news stories on ZIKV in animals, showing that 47 animal species from three orders (mammals, reptiles, and birds) have been investigated for the potential to establish a sylvatic cycle. The review aims to contribute to epidemiological studies and the knowledge on the natural history of ZIKV. The article concludes with questions that require urgent attention in epidemiological studies involving wildlife in order to understand their role as ZIKV hosts and to effectively control the epidemic.

## Introduction

Zika virus (ZIKV) is an emerging flavivirus from the same family as the West Nile (WNV), Japanese encephalitis (JEV), dengue (DENV), and yellow fever viruses (YFV) [1, 2]. ZIKV is an RNA virus, mostly transmitted to humans by bites from infected *Aedes* spp., especially *Aedes aegypti*, a highly competent and anthropophilic vector species [3] that also transmits DENV and Chikungunya virus (CHIKV) [4]. Other *Aedes* species have been implicated in ZIKV transmission, mainly in sylvatic cycles, including *Ae*. *africanus*, *Ae*. *albopictus*, *Ae*. *apicoargenteus*, and *Ae*. *furcifer* [5, 6, 7, 8].

ZIKV was first identified in 1947 in primates during a YFV study in Uganda [5]. The first reports of infected humans appeared five years later in Uganda and Tanzania [9], but the infection remained limited to equatorial regions of Africa and Asia, cycling between infective monkeys, arboreal mosquitoes, and occasionally humans [10, 11]. Mosquitoes captured annually since 1965 in Senegal have shown that ZIKV amplifies cyclically every four years, which indicates that it is the “dominant periodicity" of the ZIKV in Senegal [12]. ZIKV outbreaks in humans occurred in 2007 on the island of Yap, in Micronesia, and in Gabon [13, 6], and another outbreak occurred in 2013 in French Polynesia [14].

Recent phylogenetic and molecular studies suggest a single introduction of the ZIKV Asiatic strain into the Americas (Brazil) between May and December 2013 [15] and in February 2014 in Chile [16]. In early 2015, several patients in Northeast Brazil presented DENV-like symptoms, and molecular diagnosis revealed autochthonous ZIKV infection [17].

ZIKV has invaded a geographic area that comprises the huge Brazilian biomes, bordering on other Latin American countries. Althouse et al. [18] modeled the ZIKV transmission dynamics, estimating the numbers of primates and mosquitos needed to maintain a wild ZIKV cycle. Six thousand primates and 10,000 mosquitoes are enough to support a ZIKV transmission cycle. Based on the number of Brazilian primate species, the proximity of these and other small mammal species to urban and rural areas, and the wide distribution of *Ae*. *aegypti*, *Ae*. *albopictus*, and other mosquito genera like *Culex* [19, 20] and *Haemagogus* throughout the country, ZIKV spillover to wild primates is a potentially real scenario [21]. A wildlife cycle would launch new transmission dynamics with unknown impacts on other animal species, including humans.

This review aims to describe the available data on ZIKV infection in host animals and its relationship to biodiversity, rapid environmental changes, and the impact on human health in megadiverse Latin American countries.

## Methods

Recent advances in scientific research have emerged since ZIKV became pandemic. We searched for scientific articles and news stories on research involving ZIKV in animals using PubMed citation and index, the Fiocruz Library database, the Scopus database, and websites for news stories in the mainstream lay press.

## Results and Discussion

### Animals as ZIKV hosts

Few studies have focused on the role of animals as hosts for ZIKV. Some authors claim that there is no solid evidence of wild mammals, such as nonhuman primates (NHPs), as reservoirs for ZIKV. Meanwhile, studies have reported ZIKV antibodies in livestock like goats and sheep, rodents [22], and lions and ungulates like Artiodactyla, Perissodactyla, and Proboscidea [23]. In 1971, ZIKV antibodies were detected in primates from the Cercopithecidae family in Nigeria [24]. Several studies suggest that DENV, CHIKV, and ZIKV adapted from an ancestral enzootic transmission cycle involving NHPs and a broad spectrum of species from genus *Aedes* (*Stegomyia*, *aegypti*) as vectors in an urban/peri-urban cycle [25].

**Fig 1:**
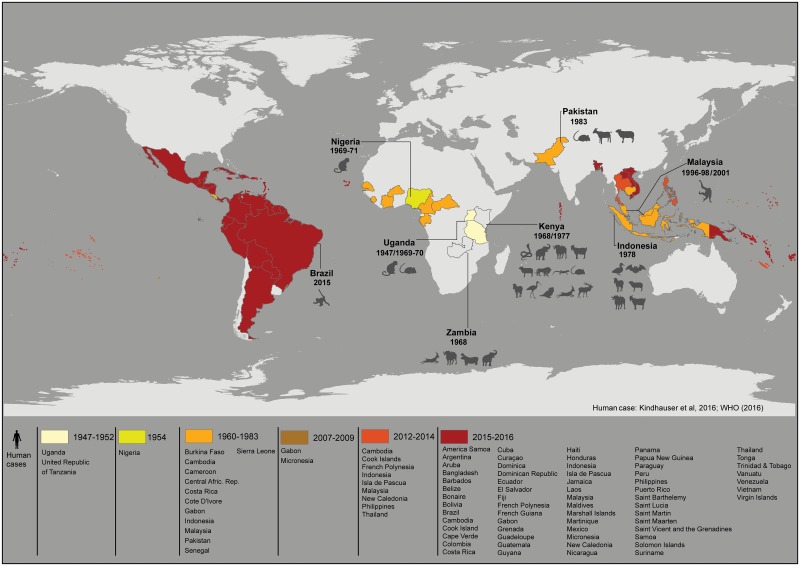
Historical time-line of ZIKV spread in humans and animals in the world. Colored countries have reported autochthonous vector-borne human cases, and those labeled with specific years and animal silhouettes have reported diagnosed cases of ZIKV in naturally infected animals. Human cases are according to references [26, 27], and the list of animal species is described in Table 1.

ZIKV infection has also been identified in other naturally and experimentally susceptible animal species (Table 1 and Fig 1). Sera from 172 domestic animals and 157 wild rodents were tested for ZIKV in Pakistan, showing that sheep, goats, some rodent species, and one human living in the same area tested positive for ZIKV antibodies [22].

**Table 1:** Chronological ZIKV natural and experimental assay infection in vertebrate hosts in the world.

		Vertebrate host							
Date report	Country	Taxonomic group		Common name	Scientific name***	Diagnostic methods for ZIKV	Type of infection	% of infection	Reference
		Order	Family						
1947	Uganda	Primates	Cercopithecidae	Rhesus monkey (sentinela)	*Macaca mulatta*	Virus isolation	N	16.7 (1/6)	[5]
1952*	London	Rodentia	Caviidae	Guineapigs	*Cavia* sp.		EA	Not clearly mentioned	[9]
1952*	London	Lagomorpha	Leporidae	Rabbit	Not mentioned	Serology	EA	100 (4/4)	[9]
1952*	London	Rodentia	Muridae	Swiss albino mice	*Mus musculus*		EA	Not clearly mentioned	[9]
1952*	London	Rodentia	Cricetidae	Cotton-rats	*Sigmodon hispidus hispidus*		EA	Not clearly mentioned	[9]
1952*	London	Primates	Cercopithecidae	Rhesus monkey	*Macaca mulatta*		EA	Not clearly mentioned	[9]
1952**	London	Primates	Cercopithecidae	Red-tailed monkey	*Cercopithecus ascanius schmidti*		EA	Not clearly mentioned	[9]
1952**	London	Primates	Cercopithecidae	Grivet monkey	*Cercopithecus aethiops centralis*		EA	Not clearly mentioned	[9]
1955*	EUA	Chiroptera	Pteropodidae	Cave bat	*Myotus lucifugus*		EA	80 (16/20)	[28]
1968	Kenya	Artiodactyla	Bovidae	Gazelle	not mentioned	HIA	N	8.3 (1/12)	[23]
1968	Kenya	Artiodactyla	Bovidae	Kongoni	*Alcelaphus buselaphus*	HIA	N	27.3 (6/22)	[23]
1968	Kenya	Carnivora	Felidae	Lion	*Panthera leo*	HIA	N	50 (1/2)	[23]
1968	Kenya	Artiodactyla	Bovidae	Wildebeest	*Connochaetes taurinus*	HIA	N	22.2 (4/18)	[23]
1968	Uganda	Primates	Cercopithecidae	African green monkey	*Cercopithecus aethiops*	HIA	N	64.6 (115/178)	[23]
1968	Uganda	Primates	Cercopithecidae	Red-tailed Monkey	*Cercopithecus ascanius*	HIA	N	21.4 (3/14)	[23]
1968	Uganda	Rodentia	Muridae	Abyssinian grass rat	*Arvicanthis abyssinicus*	HIA	N	4.6 (2/43)	[23]
1968	Zambia	Cetartiodactyla	Bovidae	African buffalo	*Syncerus caffer*	HIA	N	88.9 (8/9)	[23]
1968	Zambia	Artiodactyla	Hippopotamidae	Hippo	not mentioned	HIA	N	57.1 (20/35)	[23]
1968	Zambia	Proboscidea	Elephantidae	Elephant	not mentioned	HIA	N	46.5 (54/116)	[23]
1968	Zambia	Artiodactyla	Bovidae	Impala	*Aepyceros melampus*	HIA	N	33.3 (1/3)	[23]
1968	Kenya	Perissodactyla	Equidae	Zebra	not mentioned	HIA	N	5.5 (1/18)	[23]
1968	Kenya	Proboscidea	Elephantidae	Elephant	not mentioned	HIA	N	40.8 (31/76)	[23]
1969–1970	Uganda	Primates	Cercopithecidae	Red-tailed monkey	*Cercopithecus ascanius schmidti*	HIA and SN	N	38.1 (54/142) and 52.1 (74/142)	[7]
1969–1970	Uganda	Primates	Cercopithecidae	Colobus	*Colobus abyssinicus uellensis*	HIA and SN	N	45.4 (5/11) and 54.5 (6/11)	[7]
1969–1970	Uganda	Primates	Cercopithecidae	Mangabey	*Cercocebus albigena johnstoni*	HIA and SN	N	50 (2/4) and 75 (3/4)	[7]
1969–1971	Nigeria	Primates	Cercopithecidae	African green monkey	*Chlorocebus aethiops*	HIA and SN	N	55.5 (5/9) and 66.6 (6/9)	[24]
1969–1971	Nigeria	Primates	Cercopithecidae	Mona Monkey	*Cercopithecus mona*	HIA and SN	N	36.1 (13/36) and 41.7 (15/36)	[24]
1971	Nigeria	Primates	Cercopithecidae	Western Putty-nosed Monkey	*Cercopithecus nictitans martini*	HIA and SN	N	50 (2/4) and 25 (1/4)	[24]
1969	Nigeria	Primates	Cercopithecidae	Red-capped Mangabey	*Cercopithecus torquatus*	HIA and SN	N	100 (5/5) and 80 (4/5)	[24]
1969–1971	Nigeria	Primates	Cercopithecidae	Olive Baboon	*Papio anubis choras*	HIA and SN	N	100 (2/2) and 50 (1/2)	[24]
1969–1971	Nigeria	Primates	Cercopithecidae	Wadi monkey	*Erythrocebus patas*	HIA and SN	N	11.9 (8/67) and 59.7 (4/67)	[24]
1977	Kenya	Ciconiiformes	Threskiornithidae	African Sacred Ibis	*Threskiornis aethiopicus*	HIA	N	4.1 (2/49)	[29]
1977	Kenya	Ciconiiformes	Ardeidae	Cattle Egret	*Bubulcus ibis*	HIA	N	2.7 (1/37)	[29]
1977	Kenya	Charadriformes	Scolopacidae	Ruff	*Philomachus pugnax*	HIA	N	50.0 (1/2)	[29]
1977	Kenya	Rodentia	Muridae	African Grass Rat	*Arvicanthus niloticus*	HIA	N	4.0 (58/1446)	[29]
1977	Kenya	Rodentia	Muridae	Kaiser's Rock Rat	*Aethomys kaiseri*	HIA	N	34 (85/250)	[29]
1977	Kenya	Rodentia	Soricidae	African giant shrew	*Crocidura occidentalis*	HIA	N	3.2 (2/63)	[29]
1977	Kenya	Squamata	Lamprophiidae	Brown House Snake	*Boaedon fuliginosus*	HIA	N	40 (4/10)	[29]
1977	Kenya	Squamata	Varanidae	Common Water Monitor	*Varanus niloticus*	HIA	N	12.5 (1/8)	[29]
1977	Kenya	Cetartiodactyla	Bovidae	Goat	*Capra aegagrus*	HIA	N	0.2 (1/655)	[29]
1977	Kenya	Cetartiodactyla	Bovidae	Sheep	*Ovis aries*	HIA	N	0.7 (2/283)	[29]
1977	Kenya	Cetartiodactyla	Bovidae	Cattle	*Bos taurus*	HIA	N	0.6 (15/2324)	[29]
1978	Indonesia	Perissodactyla	Equidae	Horse	*Equus caballus*	HIA	N	20 (3/15)	[30]
1978	Indonesia	Cetartiodactyla	Bovidae	Cattle	*Bos taurus*	HIA	N	10 (4/41)	[30]
1978	Indonesia	Artiodactyla	Bovidae	Carabao	*Bubalus bubalis*	HIA	N	8 (1/13)	[30]
1978	Indonesia	Cetartiodactyla	Bovidae	Goat	*Capra aegagrus*	HIA	N	20 (7/35)	[30]
1978	Indonesia	Anseriformes	Anatidae	Duck	Not mentioned	HIA	N	4 (2/52)	[30]
1978	Indonesia	Chiroptera	Not described	Bat	Not mentioned	HIA	N	8 (6/71)	[30]
1983	Pakistan	Rodentia	Muridae	Antelope rat	*Tatera indica*	CTF	N	6.4 (3/47)	[22]
1983	Pakistan	Rodentia	Muridae	Indian desert jird	*Meriones hurrianae*	CTF	N	6.1 (2/33)	[22]
1983	Pakistan	Rodentia	Muridae	Sind rice	*Bandicota bengalensis*	CTF	N	50 (1/2)	[22]
1983	Pakistan	Cetartiodactyla	Bovidae	Sheep	*Ovis aries*	CTF	N	2.2 (1/46)	[22]
1983	Pakistan	Cetartiodactyla	Bovidae	Goat	*Capra aegagrus*	CTF	N	2.1 (1/48)	[22]
1996–1998	Malaysia	Primates	Hominidae	Western Bornean Orangutan	*Pongo pygmaeus pygmaeus*	ELISA and/or IFAT	N	8.4 (6/71)	[31]
2001	Malaysia	Primates	Hominidae	Bornean orangutan	*Pongo pigmaeus*	SN	N	8.4 (6/71)	[32]
2016	Brazil	Primates	Cebidae	Capuchin monkey	*Sapajus libidinosus*	RT-PCR	N	33.3 (3/9)	[21]
2016	Brazil	Primates	Callitrichidae	Marmoset	*Callithrix jacchus*	RT-PCR	N	26.7 (4/15)	[21]

Abbreviations: N, natural; EA, experimental assay; ELISA, enzyme-linked immunosorbent assay; RT-PCR, real-time polymerase chain reaction; HIA, hemagglutination inhibiting antibodies; IFAT, immunofluorescence antibody test; SN, serum neutralization; CTF, complement fixation test, EUA, United States of America.

*Intracerebral inoculation.

**Subcutaneous inoculation.

***The scientific names follow the exact description of the original reference and not the current taxonomic classification.

A study in Kenya in 1977 focused on the potential role of livestock (goats, sheep, and cattle) and wild vertebrates (2,424 small mammals, 1,202 birds, 18 reptiles) in maintaining arbovirus transmission. Hemagglutination inhibition assays showed that domestic animals (0.4%), wild birds (0.4%), small wild mammals (5.9%), and reptiles (27.7%) tested positive for ZIKV [29].

Serologic studies should be interpreted carefully in view of possible cross-reactions with other antigenic flavivirus, despite studies suggesting that plaque reduction neutralization test (PRNT) does not cross react and is the most specific serological test for the proper serological identification of flaviviruses [33, 34, 35].

Regarding ZIKV infection of sylvatic animals, the presence of positive animals for antibodies does not necessarily means that they are viremic, and they may not be able to transmit the virus to a mosquito, but more studies are required to properly address this issue [36]. In the case of the sylvatic cycle of YFV (also a flavivirus) in the Americas, when monkeys become infected, they present overt clinical signs and a viremia high enough to transmit virus to the mosquito vectors [37].

Unlike humans, wild mammals with ZIKV infection display few clinical signs. In a sentinel study in Uganda in 1947, primates showed only mild pyrexia. All monkeys inoculated by different routes developed neutralizing antibodies by day 14 after inoculation [5]. In the same study, Swiss mice became ill and one animal died following intracerebral inoculation [9]. Such inoculation is not a natural transmission route, and authors point out that some species of wild and laboratory rodents are resistant to some flavivirus infections due to innate genetic resistance [38].

Most primates identified as ZIKV-positive in the wild or in sentinel studies are from Old World species. Phylogenetic analysis shows that humans are more closely related to Old World primate species, especially chimpanzees and orangutans [39]. Diseases that can be transmitted between closely related species often increase the relative risk [40, 41]. NHPs thus deserve special attention because of their close relatedness to humans and potential disease exchange [42].

Favoretto et. al. [21], using real-time PCR, showed that 29% (7/24) of the New World primates, *Callithrix jacchus* and *Sapajus libidinosus*, in Ceará State in Northeast Brazil were infected with ZIKV. They also showed that the ZIKV genome sequence from monkeys was 100% similar to the ZIKV circulating in humans in South America, suggesting that primates sharing the habitat with humans could act as ZIKV hosts, as in the YFV sylvatic cycle in Brazil.

Besides the use of primates as sentinels in ZIKV studies, some experimental work has been performed with other mammals. Cotton-rats, guinea pigs, and rabbits showed no clinical signs of infection after intracerebral inoculation [9]. An experiment in 1955 aimed to determine the susceptibility of cave bats to ZIKV and showed that these bats are susceptible to ZIKV by intraperitoneal, intradermal, intracerebral, and intrarectal exposure, but not by intranasal exposure [28].

Barr et al. [43] infected cell cultures from different animal species with ZIKV and showed that 17 were susceptible to the virus, developing a cytopathic effect seven days post infection. Some of the cell cultures were from domestic animals and others from Old World wild primates, while nine were from wild animals species found in the Americas: *Tabarida brasiliensis*, *Sylvilagus floridanus*, *Urocyon cinerorgeneus*, *Odocoileus hemionus*, *Procyon lotor*, *Didelphis virginiana*, *Dasypus novemcinctus*, *Marmota monax*, and *Neovison vison*. Most of these animals are peri-domestic and sympatric to mosquito vectors. The authors also argued that with sufficiently high viremia, these animals could serve as hosts. However, they also indicated that the virus strain used in the experiment lacks some characteristics of the ZIKV currently circulating in the field, and that the virus in the laboratory does not mirror natural infection.

Public policy and elimination efforts in the Americas are based mainly on vector control and personal protection measures, so the high number of wild species with the potential to establish a sylvatic cycle would make elimination extremely difficult, if not impossible [18]. We thus need studies on ZIKV in wild and domestic animals in the Americas, both to understand their potential role as hosts in the natural cycle and to target surveillance for enzootic ZIKV transmission.

### Biodiversity, animal hosts, and diseases

Human health relates closely to environmental health, defined here as the relationship between the health of domestic animals, wildlife, and the environment. Most etiological agents (60.3%) circulate between animals and humans, and 71.8% of emerging diseases are caused by pathogens originating in wildlife [44]. A recent study associated 2,107 etiological agents with diseases in humans and animals [45].

Recent efforts by the Convention on Biological Diversity and the World Health Organization have addressed scientific and political discussions on the relationship between human health and biodiversity. Such relationships include global concern over the importance of emerging zoonotic diseases originating in wildlife. Environmental changes, including loss of biodiversity, can favor emerging diseases originating from wildlife and act as the source of selective forces in new genetic variations, leading to spillover and infecting humans [46]. This justifies actions to improve knowledge on biodiversity and pathogens and to monitor them to anticipate problems.

The current ZIKV epidemic in Brazil requires understanding of the role of mammals, especially primates, in viral transmission to humans, especially when this interface occurs in fragmented forest areas, as described by Favoretto et al. [21]. Such areas are usually bordered or surrounded by farmland and human settlements and by dense urban and unstructured areas that can increase contact between humans, wildlife, and domestic animals and occasionally promote disease spillover [47, 48]. Wild animals, especially primates, can thus be considered sentinels for pathogens of human health concern [48, 49]. ZIKV is an example of spillover, because this virus adapted from an ancestral transmission cycle involving NHPs to an urban/peri-urban cycle, with humans as the main host.

Brazil is a megadiverse country with 357 million hectares of tropical forest and other highly biodiverse biomes [50]. Not surprisingly, Brazil has more primate species than any other country. Its 53 species account for 27% of the world’s primates [51].

Some NHP species occupy urban forests due to habitat fragmentation and have close contact with humans and domestic animals. Examples include primates from the Callitrichinae (*Callithrix*, *Leontopithecus*, and *Saguinus*), Cebinae (*Cebus*), and Atelidae families (*Alouatta* and *Brachyteles*) [52]. Favoretto et al. [21] were the first to report ZIKV in NHPs in Northeast Brazil, highlighting that these New World primates can act as potential ZIKV hosts in the Americas. Many questions remain unanswered. Does ZIKV impact the health of NHPs? Are NHPs living in urban fragments of forest more prone to ZIKV infection than those in preserved areas? Can naturally infected neotropical primates transmit ZIKV to mosquito vectors and thus help keep the virus circulating in the Americas?

Barr et al. [43] demonstrated the feasibility of infection in cell cultures from other mammalian species like carnivores, armadillos, rodents, and bats, thus raising the possibility of a transmission network shaped by biological and ecological factors. These factors include vector and host density and behavior, virulence, viral load, immunity, genetic variation, climate change, competition between biological communities, and anthropogenic forces like urbanization, sanitation, limited access to health services, poverty, and mistreatment of animals [38].

Considering the current epidemiological scenario with simultaneous circulation of the arboviruses ZIKV, DENV, and CHIKV and the fact that Brazil has a large NHP population, there is an urgent need to answer these questions to evaluate the impact of diseases like Zika on the NHP population in Brazil and elsewhere in the Americas. YFV, another flavivirus that circulates in a sylvatic cycle in the Americas, has a great impact on primate populations, especially those of genus *Alouatta* [53], which exhibit disease signs after infection and act as sentinel primates for viral circulation and for implementation of control measures like human vaccination campaigns.

The pandemic ZIKV strain differs significantly from the African strain mainly in two regions of the genome. These acquired genetic markers increase its fitness for replication in the human host [4]. Whether these mutations also alter the infectivity in NHPs remains to be determined. The role of wild primates and other mammals in ZIKV epidemiology thus requires urgent investigation.

The complex epidemiological panorama currently experienced in several countries of South America, with the co-circulation of three arbovirus, ZIKV, DENV, and CHIKV, of high impact on public health, highlights the importance of a robust epidemiological surveillance. During 2014, two strains of CHIKV were introduced in Brazil: the Asian and the African (East/Central/South Africa [ECSA]) strains, both transmitted by *Ae*. *aegypti*.

As seen during the 2005 CHIKV outbreak in La Réunion Island, where the predominant mosquito species was *Ae*. *albopictus*, the viruses quickly acquired an E1-A226V mutation, increasing viral fitness to infect *Ae*. *albopictus*, which became the principal vector [54]. The Brazilian CHIKV strains analyzed so far did not display mutations that increase CHIKV transmissibility and persistence in *Ae*. *albopictus* [55]. However, the elevated density and wide distribution of *Ae*. *albopictus* in Latin America warns the risk of the ECSA strain adapt to this vector [56]. Moreover, the abundance of naïve primate (and maybe other small mammals) species and culicids species in South American forests creates the scenario for the establishment of an enzootic cycle, as seen in Africa and Asia, where there is evidence of a sylvatic CHIKV transmission cycle involving NHPs and mosquitoes [57].

Another relevant issue is the development of diagnostic tests for the detection of ZIKV infection in wild mammals, enabling unequivocal results without cross-reactivity with other flavivirus infections.

## Final Comments and Research Perspectives

Despite the growth of epidemiological knowledge in the last century, health interventions still mainly react to emergency events involving specific diseases in the human population, with some mitigation efforts [46]. The current ZIKV epidemic is no exception. We cannot expect to completely block the emergence of diseases, considering vector spread due to our limited capacity to reverse climate change, the globalization of goods and people, and our mode of production and consumption of natural resources. This situation is particularly paradoxical in megadiverse countries like Brazil.

The driving forces in the spread of diseases apply to the ZIKV epidemic, including anthropogenic activities, climatic change, intense human movement, loss of biodiversity, habitat destruction, land use change, introduction of invasive species, urban development, lack of knowledge on the role of animals in maintaining the sylvatic cycle, clinical manifestations, and wildlife trafficking [46].

We need to understand the diversity of pathogens in nature and correlate them with biological communities, pathogenic and genetic characteristics, and anthropic impacts in areas where disease transmission occurs. DENV is a good example of how a combination of environmental changes, genetic characteristics, and human mobility propels the spread of viruses in Brazil. A new lineage of DENV entered in the country through Caribbean through the northern/northeast and spread rapidly to the rest of Brazil, especially through the aerial transportation of humans and/or mosquito vectors [58]. In parallel, this example allows us to suggest that the spread of ZIKV to other biomes in the Americas and outside Brazil may also be related to these factors, and that these should be highlighted.

The ZIKV epidemic illustrates the importance of monitoring and predicting the pathogens arising from wild animals and biodiversity. Based on the above and the results of other studies, we pose several questions and hypotheses that emerge from this discussion and that require investigation:

1. What other wild animals besides primates could be infected by ZIKV in Americas? What is their role in maintaining and transmitting the virus to mosquito vectors? Which species can act as hosts?
2. Does the virus circulate at higher levels in wild animals inhabiting forest fragments adjacent to urban areas? What role do these animals play in maintaining the virus in areas close to humans?
3. Which wild hosts help keep the virus circulating in the Americas?
4. Do neotropical primates play a special role in the ZIKV epidemic?
5. Does ZIKV impact wild animal populations and biodiversity? Does it cause disease and mortality in these animals?

Infectious diseases have important implications for animal and human health and biodiversity. Public health and biodiversity needs are misaligned and need to be rebalanced.

Rather than merely attacking and solving epidemic situations, as in the current ZIKV global health emergency, we need to predict and prevent future emerging diseases. Studies of wild hosts are troublesome and costly, especially when they require long-term monitoring. Funding also needs to be targeted for these studies. Future laboratory, field, and eco-epidemiological research should focus on wildlife hosts to elucidate their role in ZIKV epidemiology in the Americas and enhance the epidemic’s control.

**Key Learning Points**

- A remarkable diversity of wildlife species involving three orders, namely mammals, reptiles, and birds, totaling 47 different species, were investigated for their potential to establish a sylvatic ZIKV cycle.
- A study showed that New World primates in Northeast Brazil were infected and that the ZIKV genome sequence in monkeys was 100% similar to the ZIKV circulating in humans in South America. Studies are thus needed on ZIKV in wild and domestic animals in the Americas, both to understand their potential role as hosts in the natural cycle and to target surveillance for enzootic ZIKV transmission.
- Environmental changes, including loss of biodiversity, can favor emerging diseases originating from wildlife and act as selective forces in new genetic variations leading to spillover and infection in humans. This justifies actions to improve knowledge on biodiversity and pathogens and to monitor them to anticipate problems.
- Driving forces in the spread of diseases apply to the ZIKV epidemic, including anthropogenic activities, climatic change, intense human movement, loss of biodiversity, habitat destruction, land use change, introduction of invasive species, urban development, lack of knowledge on the role of animals in maintaining the sylvatic cycle, clinical manifestations, and wildlife trafficking.
- Future laboratory, field, and epidemiological research should focus on wildlife hosts to elucidate their role in ZIKV epidemiology in the Americas and enhance the epidemic’s control.

**Top Five Papers**

1. Favoretto S, Araújo D, Oliveira D, Duarte N, Mesquita F, Zanotto P, et al. First detection of Zika virus in neotropical primates in Brazil: a possible new reservoir. In press. 2016. BioRxiv.
2. Dick GW, Kitchen SF, Haddow AJ. Zika virus. I. Isolations and serological specificity. Trans R Soc Trop Med Hyg. 1952; 46: 509–520.
3. Althouse BM, Vasilakis N, Sall AA, Diallo M, Weaver S, Hanley KA. Potential for Zika virus to establish a sylvatic transmission cycle in the Americas. In press. 2016. BioRxiv.
4. Henderson BE, Hewitt LE, Lule M. Serology of wild mammals. In: Virus Research Institute 409 Annual Report. East African Printer, Nairobi, Kenya. 1968, pp. 48–51.
5. Karesh WB, Formenty P. Infectious diseases. In: WHO. Connecting Global Priorities: Biodiversity and Human Health. A State of Knowledge Review, 2015, pp.28.
